# Overexpression of *Nicotianamine Synthase* (*AtNAS1*) Increases Iron Accumulation in the Tuber of Potato

**DOI:** 10.3390/plants11202741

**Published:** 2022-10-17

**Authors:** Manrong Zha, Xin Li, Rui Li, Jing Huang, Jinping Fan, Jing Zhang, Yan Wang, Cankui Zhang

**Affiliations:** 1 College of Biology Resources and Environmental Sciences, Jishou University, Jishou 416000, China; 2 Department of Agronomy, Center for Plant Biology, Purdue University, West Lafayette, IN 47907, USA; 3 Beijing Key Laboratory of Growth and Developmental Regulation for Protected Vegetable Crops, College of Horticulture, China Agricultural University, Beijing 100193, China; 4 College of Agronomy, Sichuan Agricultural University, Chengdu 611130, China; 5 Department of Horticulture and Landscape Architecture, Northeast Agricultural University, Harbin 150030, China; 6 Department of Plant Biology, Cornell University, New York, NY 14853, USA

**Keywords:** potato, tuber, Fe content, *AtNAS1*, phloem

## Abstract

Iron (Fe) deficiency is a global health problem, especially in underdeveloped countries. Biofortification with genetic engineering methods has been used to improve Fe nutrition in a number of crops. Various steps, e.g., uptake, distribution, and storage, involved in Fe homeostasis have been manipulated to increase the Fe concentration in the edible portions of plants. Nicotianamine (NA) is an important metal ion chelator in plants. It promotes the mobility of Fe and decreases cellular Fe toxicity. Increasing the Fe content in crops by promoting NA synthesis could help decrease human diseases associated with Fe deficiency. In the present study, *Arabidopsis thaliana* nicotianamine synthase 1 (*AtNAS1*) was overexpressed in potato (*Solanum tuberosum*, *St*) under the control of the cauliflower mosaic virus 35S promoter. Transgenic plants had a significantly increased amount of Fe in tubers (52.7 µg/g dry weight, 2.4-fold the amount in wild-type tubers), while no differences in plant phenotype or yield were detected between transgenic and wild-type plants. The expression of genes involved in root mineral uptake and homeostasis, such as *StYSL1*, *StIRT1*, *StFRO1*, and *StNAS*, was also altered in the roots and leaves of the transgenic plants. Our results demonstrate that the manipulation of Fe chelation is a useful strategy for Fe nutrition improvement, and the increased Fe accumulation in tubers of transgenic potato plants is most likely caused by the increased movement of Fe from the leaf to the tuber.

## 1. Introduction

Iron (Fe) is an essential micronutrient required for the survival and proliferation of all plants [1]. It is involved in chlorophyll (Chl) biosynthesis, photosynthetic/respiratory electron transport processes, and protection against radical oxygen species, and is also a component of heme [2,3]. Fe deficiency is now a widespread agricultural problem, leading to low yield and poor nutritional quality of crops—over one-third of the world’s soils are considered Fe-deficient, and the availability of soluble Fe to plants is also restricted in alkaline soils [4]. Fe is also critical to human health. Lack of Fe may result in adverse health outcomes, and even a slight Fe deficiency can lead to deficits in cognitive function in children [5]. Increasing the Fe level in food crops is an important strategy to decrease the diseases caused by Fe deficiency.

Plant Fe deficiency is characterized by the yellowing of young leaves, the thickening of root tips, and amplified root hair formation and elongation [6]. Plants have evolved two Fe-absorption strategies to cope with Fe shortage and recover Fe from the soil. The first, reduction (strategy I), is found in all dicots and non-graminaceous monocots, and involves the conversion of ferric (Fe^3+^) to ferrous (Fe^2+^) iron by ferric-chelate reductase (FCR) and transport of Fe^2+^ into roots by Fe-regulated transporter 1 (IRT1) [7,8]. The second strategy, chelation (strategy II), is unique to gramineous plants; in this system, phytosiderophores are released into the rhizosphere to chelate Fe^3+^ and are then transported into roots by the YELLOW STRIPE 1 (YSL1) transporter [9,10,11]. To address how plants control Fe homeostasis, plant responses to Fe deficiency have been widely studied, particularly in *Arabidopsis thaliana*. For example, an intricate network of basic helix–loop–helix (bHLH) transcription factors has been highlighted, which plays crucial roles in positively regulating various Fe-deficiency-inducible genes, e.g., *IRT1*, *FRO2*, and *AHA2/7* [12,13,14]. Interestingly, Fe itself plays a signaling role and is regarded as a positive regulator of the local signal for iron uptake activity [15].

Understanding the molecular mechanisms related to Fe homeostasis helps implement transgenic approaches for Fe nutrition improvement. In most previous research, genes responsible for Fe uptake and storage were used, individually or in combination, to produce transgenic plants with significantly increased Fe content in plant organs [16,17,18,19,20]. However, most of these studies were focused on cereal crops such as rice or model species such as *Arabidopsis*. Little progress has been made in tuber-producing crops, which are considered important staple foods.

Tubers of potato and cassava are full of carbohydrates but low in Fe and other metal nutrients. Potato (*Solanum tuberosum*, *St*) plants follow a reduction-based strategy (strategy I) for Fe uptake, which is different from other major crops such as rice, wheat, and maize [21,22]. The tuber is a specialized stem known as the stolon and is not formed from the root. When Fe is absorbed by the basal roots of potato from soils, it is transported to the shoot through the xylem. This process is driven by leaf transpiration [22]. During transport, some Fe is exchanged into the phloem and moved to tubers, driven by the source-to-sink movement of sucrose via phloem [2,23,24,25].

Nicotianamine (NA) is a non-proteinogenic amino acid, first found in tobacco, that exists in all higher plants [26,27,28]. It plays a crucial role in the chelating, transport, and homeostasis of Fe and other heavy metals [27]. NA is required for the movement of Fe in the phloem [29]. Synthesis of NA occurs via the trimerization of *S*-adenosyl-L-methionine in a process catalyzed by the enzyme nicotianamine synthase (NAS) [30]. NAS-encoding genes have been recognized as valuable targets for generating micronutrient-enriched crops [16,17,31]. *NAS* genes have been overexpressed using strong constitutive promoters, which can increase Fe approximately 2-fold in grains of rice and wheat [17,32].

In this study, our objective was to test whether manipulation of the expression of *NAS* and the associated Fe movement leads to improved Fe content in the tuber of potato, a crop that is significantly different from cereals.

## 2. Results

### 2.1. Generation of 35S::AtNAS-Overexpression Potato

**Figure 1:**
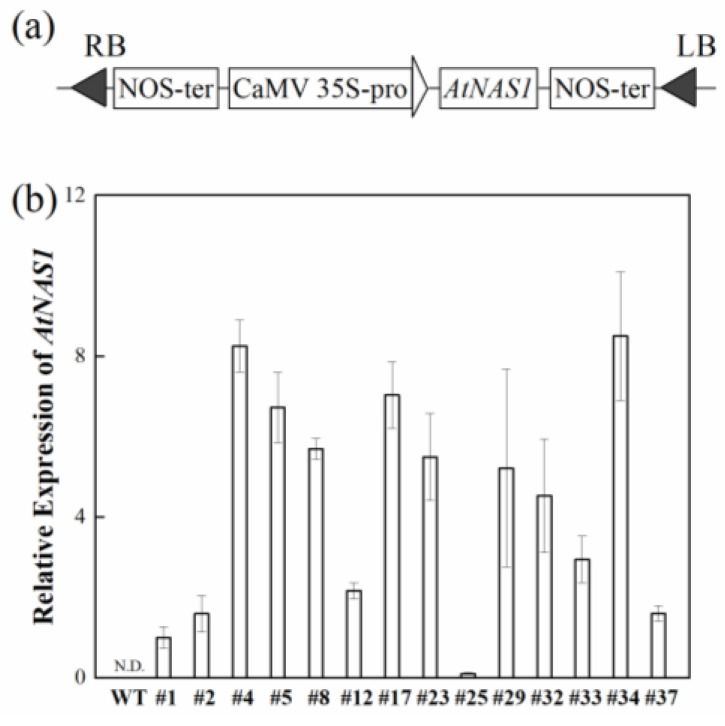
Confirmation of *35S::AtNAS1* transgenic potato. (**a**) Schematic representation of the T-DNAs used for constitutive overexpression of the *AtNAS1* gene. RB, right border; NOS-ter, nopaline synthase terminator; CaMV 35s pro, dual *CaMV* 35S promoter; *AtNAS1*, coding sequence of *AtNAS1* (975 bp); LB, left border. (**b**) Expression of *AtNAS1* in independent transgenic potato plants and wild-type (WT) plants, determined by quantitative real-time PCR analysis. *Actin* (XM_006350963) was used as an internal control to normalize the expression of the transgene. The data represent means from three biological replicates. Error bars = standard deviation (SD).

The *AtNAS1* gene encodes a major NAS in *Arabidopsis*. It was cloned into the pBI121 vector under the control of the 35S promoter (*35S::AtNAS1*) (Figure 1a). The expression of *AtNAS1* in the leaves of transformed potato plants was measured by qRT-PCR. All transgenic lines showed some expression of *AtNAS1*, although the relative expression level varied (Figure 1b). Because of the high expression of *AtNAS1* in transgenic lines #4 and #34, these lines were used in further analyses.

### 2.2. AtNAS1 Overexpression Had No Influence on Shoot Growth and Tuber Yield

**Figure 2:**
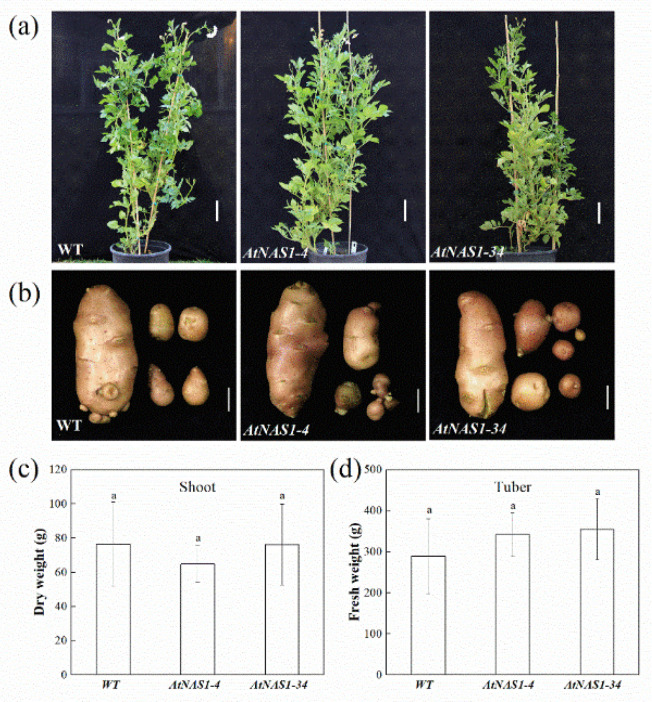
Phenotype of *AtNAS1*-overexpressing potato and WT plants grown in a greenhouse. (**a**) Images of transgenic potato grown in soil. (**b**) Tubers of transgenic and WT potato. Shoot dry weight (**c**) and tuber fresh weight (**d**) of transgenic and WT potato. The data represent means from three biological replicates. a in the picture represents significant difference. Error bars = SD. Student’s *t*-test was used to examine statistical significance, *p* < 0.05.

To determine whether the overexpression of *AtNAS1* affected the general plant phenotype, transgenic lines #4 and #34 and wild-type (WT) plants were planted in a greenhouse. Three months after transplantation into soil, the weights of the shoots and tubers of *35S::AtNAS1* and WT potato plants were measured. No significant difference was observed between *35S::AtNAS1* plants and the WT in either shoot architecture or tuber size (Figure 2a,b). The dry weights of potato shoots and fresh tuber weights showed some differences, but they were not statistically significant (Figure 2c,d). Based on these results, we concluded that overexpression of *AtNAS1* in the potato did not lead to abnormal phenotypes in the transgenic plants.

### 2.3. Constitutive AtNAS1 Expression Alters Fe and Zn Accumulation in Potato Tubers

**Figure 3:**
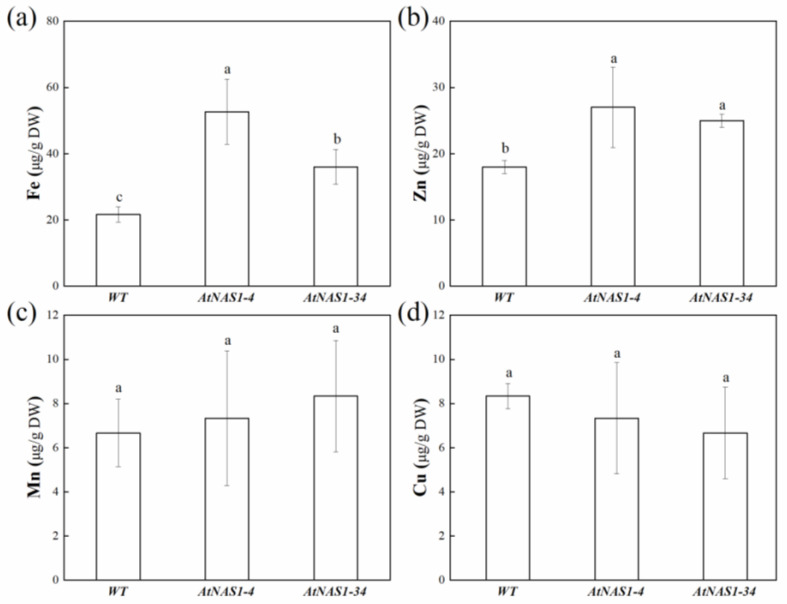
Elemental concentrations in tubers from *AtNAS*-overexpression potato and WT plants. Fe (**a**), Zn (**b**), Mn (**c**), and Cu (**d**) contents of tubers from transgenic and WT plants. The data represent means from three biological replicates. a, b, c in the picture represents significant difference. Error bars = SD. Student’s *t*-test was used to examine statistical significance, *p* < 0.05.

Because NA has been reported to be involved in the movement of multiple metals, we measured the contents of Fe, Zn, Mn, and Cu in tubers. The Fe and Zn concentrations were significantly higher in the tubers of *AtNAS1*-overexpression potatoes than in tubers from WT potatoes; the impact on Fe was more dramatic than that on Zn. The tuber Fe concentrations of the two transgenic lines were estimated to be 52.7 ± 9.8 and 36 ± 5.3 µg/g dry weight 2.4 and 1.7-fold that in WT tubers, respectively (Figure 3a). The Zn concentrations of the same tubers were 27 and 25 µg/g FW, 1.5 and 1.4-fold more than that in the WT, respectively (Figure 3b). No differences were observed between the transgenic and WT tubers in Mn or Cu concentrations (Figure 3c,d).

### 2.4. The Photosynthesis Rate and Chl Content in Potato Leaves Were Not Changed

**Figure 4:**
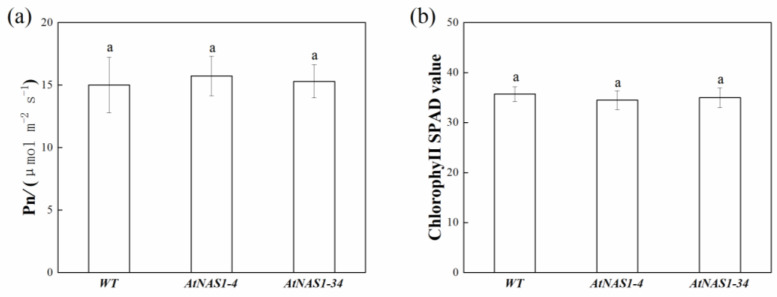
Analysis of (**a**) the photosynthesis rate and (**b**) the chlorophyll content in leaves of *AtNAS1*-overexpression and WT potato plants. a in the picture represents significant difference. Error bars = SD. Student’s *t*-test was used to examine statistical significance, *p* < 0.05.

To assess whether *AtNAS1* overexpression altered the biosynthesis of Chl in potato leaves, the photosynthesis rate and Chl concentration (SPAD value) were measured. As shown in Figure 4, no significant difference was detected between WT and transgenic potato plants, indicating there are no negative effects of *AtNAS1* overexpression on the Chl content or photosynthesis in potato leaves. These results are consistent with the lack of changes in shoot and tuber biomass accumulation in the *AtNAS1*-overexpression lines.

### 2.5. Expression of Genes Related to Fe Uptake and Homeostasis Was Altered

**Figure 5:**
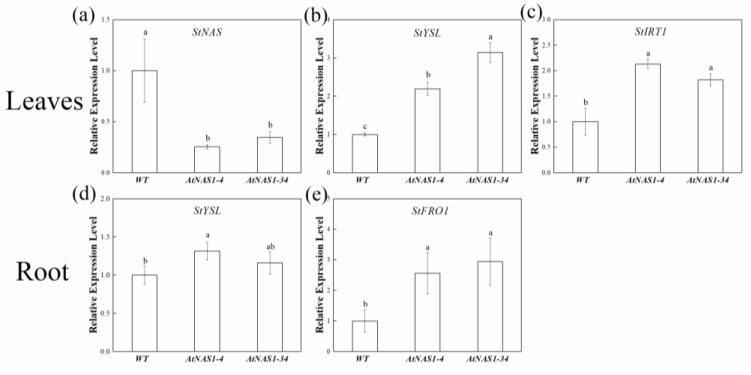
Analysis of mineral uptake and transport gene expression in potato. (**a**) *StNAS*, (**b**) *StYSL*, and (**c**) *StIRT* transcript accumulation in the leaves of transgenic and WT potato plants. (**d**) *StYSL* and (**e**) *StFRO2* transcript accumulation in the roots of transgenic and WT potato plants. *Actin* (XM_006350963) was used as an internal control to normalize the expression of the transgene. The data represent means from three biological replicates. a, b, c in the picture represents significant difference. Error bars = SD. Student’s *t*-test was used to examine statistical significance, *p* < 0.05.

Because the increased tuber Fe content in the transgenic potato plants did not lead to decreased Fe content in shoots (Supplemental Figure S1), we hypothesized that other plant systems related to Fe and Zn homeostasis might be changed in the transgenic potato plants. The expression of genes involved in root mineral uptake and homeostasis, including *IRT* (zinc/iron-regulated transporter-related protein), *FRO* (ferric reductase oxidase), and *YSL*, was determined in our study (Figure 5). The endogenous expression of *StNAS* was also measured. To our surprise, the expression level of the *StNAS* gene was decreased in transgenic potato leaves (Figure 5a). This indicated that a homeostasis mechanism might have been initiated in response to the increased Fe transport ability derived from the overexpression of *AtNAS1* in transgenic potato plants. Downregulation of the endogenous *StNAS* could decrease the scale of the alteration of Fe movement due to the overexpression of *AtNAS*. In addition, a remarkable induction of *StYSL1*, *StFRO*, and *StIRT1* was observed in the transgenic potato leaves (Figure 5b–e).

## 3. Materials and Methods

### 3.1. Plant Materials and Growth Conditions

Plants of the French potato variety *Désirée*, a model variety for potato species, were grown in a propagation mix (Sun Gro Horticulture) in a greenhouse under a 14-h light/10-h dark regime at 25–28 °C. These soils have Fe concentration at ~22 ppm and 3.5 mmho/cm EC. Roots and leaves were collected 3 months after transplanting from tissue culture medium to soil. These tissues were snap-frozen in liquid nitrogen and then stored at −80 °C before RNA extraction. Potato tubers were harvested at the end of the growth season, sliced, and dried before elemental analysis was conducted.

### 3.2. Plasmid Construction, and Transformation of Potato

The coding region of *AtNAS1* without the stop codon was amplified by PCR from cDNA with the forward primer 5′-ATGGCTTGCCAAAACAATCT-3′ and the reverse primer 3′-TTACTCGATGGCACTAAACT-5′, to yield a 1146-bp fragment containing the *AtNAS1* cDNA. The PCR fragment was cloned behind a double cauliflower mosaic virus (*CaMV*) 35S promoter in the binary vector pBI121 [33]. The complete vector was introduced into the potato via *Agrobacterium*-mediated transformation, as previously described [34].

### 3.3. RNA Extraction and Quantitative RT-PCR

Total RNA was extracted from the roots and leaves of potato plants using an E.Z.N.A.^®^ Total RNA Kit I (Omega Bio-tek, Norcross, GA, USA). A 5-μg aliquot of total RNA was used for library preparation, as previously described [33]. Sequencing was conducted on an Illumina HiSeq 2500 instrument at the Genomics Resources Core Facility of Weill Cornell Medical College.

Total RNA samples were treated with RQ1 DNase (Promega, Madison, WI, USA) for 30 min to remove genomic DNA, and then converted into cDNA using iScript™ Reverse Transcription Supermix (Bio-Rad, Hercules, CA, USA). Quantitative real-time PCR (qRT-PCR) was conducted in a CFX Connect Real-Time System with iTaq Universal SYBR Green Supermix (Bio-Rad). The thermal conditions were 95 °C for 3 min, then 40 cycles of 95 °C for 15 s and 60 °C for 60 s, followed by melt curve analysis to verify the specificity of amplification. The ΔΔCt method was used to analyze expression levels with the potato *actin* gene (XM_006350963) as an internal control.

### 3.4. Elemental Analysis

Metal contents were measured as described by Lee et al. [35]. After drying for 2 days at 70 °C, samples were digested in 1 mL of 11 M HNO_3_ for 3 days at 180 °C. After dilution, metal concentrations in the samples were determined by atomic absorption spectrometry (SpectrAA-800; Varian, Palo Alto, CA, USA) or by inductively coupled plasma mass spectrometry (ELAN6100; Perkin-Elmer, Norwalk, CT, USA).

### 3.5. Determination of Photosynthesis Rate and Chlorophyll Concentration

An Li-6400 portable photosynthesis machine (LICor Inc, Lincoln, NE, USA) was used to measure the net photosynthesis rate according to the manufacturer’s instructions, with a measuring time between 10 am and 12 pm. The SPAD value was determined using a SPAD-502 chlorophyll meter (Konica Minolta Inc., Tokyo, Japan). To lower the measurement error, 5–10 leaves from each potato plant were measured, and the average value was taken as the SPAD value.

### 3.6. Statistical Calculations

Results were analyzed using SPSS software v16.0 for Windows. Data from each sampling event were analyzed separately. Means were tested with Student’s *t*-test, and the significance level was set at *p* < 0.05.

## 4. Discussion

It has been estimated that more than 1.2 billion people are at severe risk of low dietary Fe [36]. Fe deficiency is the main cause of anemia [37]. It also results in poor pregnancy outcomes, impaired cognitive development, lower immunity, and compromised mental health [37,38]. Although Fe can be added as a food supplement to alleviate the deficiency, this approach can be costly, and it is not always available in some underdeveloped countries. Biofortification, in which conventional breeding or genetic engineering is used to increase the Fe concentration in the edible parts of plants, has become a popular way to improve the Fe nutrition value of various crops [39].

In recent decades, the expression of genes involved in various steps of Fe homeostasis, including uptake and storage, has been manipulated to increase Fe levels in a number of plants [17,32,39,40,41,42]. For example, the overexpression of the Fe uptake gene *Oryza sativa OsIRT1* led to a 13% increase in Fe in seeds [40], and the overexpression of the soybean Fe storage gene *ferritin* in rice led to a twofold increase in the Fe content in the seeds [43]. In addition to uptake and storage, the distribution of Fe, mediated by NA, is another step that has been manipulated for biofortification purposes. Increasing the expression of *NAS* has been shown to be useful in generating Fe-rich seeds of cereal plants [16,18,32,44]. For example, overexpression of *OsNAS3* resulted in 2.5-fold more Fe in polished rice [16]. A similar improvement was also observed when *HvNAS1* (encodes a major barley *NAS*) was overexpressed in polished rice [44]. Expression of *OsNAS2* in wheat increased the Fe concentration in white flour by 1.5-fold [32]. However, most of these previous studies focused on cereal crops, and very little work has been conducted on tuber-producing crops. In one recent work, the *HvNAS1* gene was overexpressed in sweet potato, and this manipulation resulted in twofold more iron in storage roots [18]. However, sweet potato is a tuberous root that belongs to the morning glory family [45], whereas regular potato is an underground stem tuber that belongs to the nightshade family [46]. In the present study, we generated transgenic potato plants that showed improved Fe content in the tubers without negatively impacting the plant phenotype or yield when compared with WT plants.

The potato tuber is a specialized stem that arises from the underground organ known as the stolon [47]. The main storage composition in potato tubers is starch [48]. It has been recognized that movement of shoot-to-stolon sucrose via the phloem is not only essential in supplying the substrate for starch biosynthesis in tubers [49], but it is also the driving force for the movement of minerals, e.g., Fe, from aboveground to underground tissues. The movement of Fe in phloem requires chelation by NA, and our results suggest that *AtNAS1* overexpression may increase the chelation process and enhance Fe transport from shoot-to-tuber. Our work demonstrates the possibility of biofortifying potato with Fe without a yield penalty. The slightly increased Zn content in tubers of the transgenic plants showed that the movement of Zn in potato phloem may also involve chelation by NA, and the increase in this chelation process can simultaneously improve the nutrient levels of both Fe and Zn. This finding may be extended to other tuber-producing crops such as cassava and yam; these are staple crops in developing countries and are similar to potato.

Fe participates in several cellular activities in plants, such as photosynthesis and respiration. Fe deficiency can affect Chl synthesis. It is important to understand whether growth and biomass accumulation are affected in a biofortified crop. An interesting result from our analyses was that *AtNAS1* overexpression increased Fe content in the tuber, but did not affect the Chl concentration, photosynthesis rate, or Fe content in potato leaves. These data suggest that Fe uptake from the rhizosphere and root-to-shoot transport might be enhanced to compensate for the increased export of Fe from leaves to tubers. This is consistent with our findings that the expression of Fe uptake and redistribution genes, e.g., *StYSL*, *StFRO1*, and *StIRT1*, was increased in the transgenic potato plants. In strategy I plants, e.g., potato, Fe^3+^ is reduced to Fe^2+^ by FRO1 (ferric reductase oxidase 1) before it is transported from soil to root via IRT1 [50,51]. Plant roots excrete protons to the rhizosphere via a proton ATPase pump, which facilitates the reduction of Fe^3+^ to Fe^2+^, catalyzed by FROs [52]. The increased expression levels of *FRO1* and *IRT1* genes in the transgenic potatoes here indicated that both the reduction and uptake processes were more active in the transgenic plants. *YSL* is expressed in vascular tissues and is responsible for the distribution of Fe [11]. The increased expression of this gene indicates that a more active distribution system has been activated to be compatible with the increased amount of Fe taken up by the roots. Our results showed that the improved Fe nutrition in the *AtNAS1*-overexpressing potato involved synergistic activities of other steps, e.g., Fe reduction and uptake, to satisfy the enhanced Fe distribution to tubers. An interesting observation in the transgenic plant was the downregulation of the native potato *NAS1* gene expression. This indicated that a homeostasis mechanism might have been initiated in response to the increased Fe transport ability derived from the overexpression of *AtNAS1* in transgenic potato plants.

## 5. Conclusions and Indications for the Future

To improve the Fe content in tuber-producing carbohydrate crops more significantly, genes involved in other steps of Fe metabolism can be simultaneously manipulated with genes involved in Fe chelation and transport. For example, ferritin is an important protein for Fe storage. The overexpression of genes associated with ferritin has been shown to increase the Fe content in rice, maize, and wheat [20,41,53]. In cassava, the overexpression of an Fe transporter and ferritin led to a much higher increase in Fe content than the manipulation of the Fe transporter gene alone [18]. It will be interesting to explore the scale of Fe content improvement in potato if genes involved in Fe uptake, chelation, and storage are manipulated simultaneously. Another factor that needs to be considered in future molecular biofortification efforts is the use of tissue-specific promoters. For example, it is known that the shoot-to-tuber movement of Fe occurs in the phloem. It will be interesting to explore whether a strong phloem-specific promoter leads to increased Fe content in tubers if fused in front of the Fe transporter or NA biosynthesis gene.

## Supplementary Materials

The following supporting information can be downloaded at: https://www.mdpi.com/article/10.3390/plants11202741/s1. Figure S1: Fe concentration in the leaves of wild type and two transgenic plants.
