# Supplementary material for: Overexpression of Nicotianamine Synthase (AtNAS1) Increases Iron Accumulation in the Tuber of Potato
Source: Plants (Basel). 2022 Oct 17;11(20):2741. doi: 10.3390/plants11202741 (PMC9607507; doi:10.3390/plants11202741)
Supplement: Supplementary file 1 [file plants-11-02741-s001.zip › plants-1943691-supplementary.pdf]

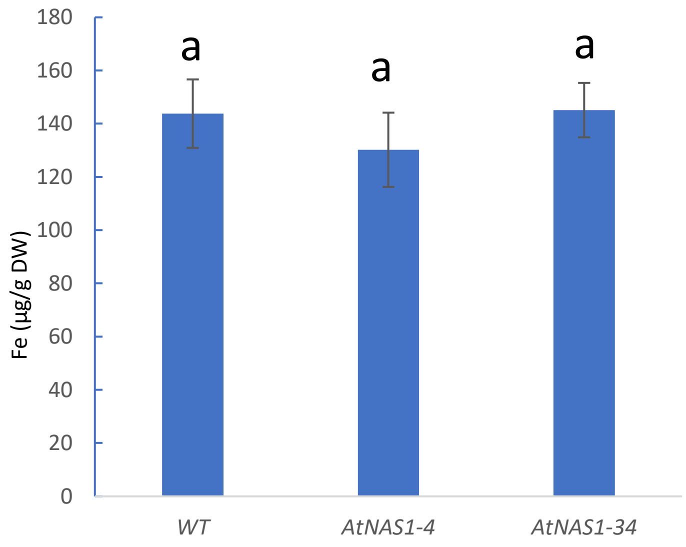

Supplemental Figure S1. Fe concentration in the leaves of wild type and two transgenic plants. The data represented means from three replicates. Error bars=SD. Student's t-test was used to examine statistical significance. $P<0.05$.
